# ^13^C Metabolic Flux Analysis Indicates Endothelial Cells Attenuate Metabolic Perturbations by Modulating TCA Activity

**DOI:** 10.3390/metabo11040226

**Published:** 2021-04-07

**Authors:** Bilal Moiz, Jonathan Garcia, Sarah Basehore, Angela Sun, Andrew Li, Surya Padmanabhan, Kaitlyn Albus, Cholsoon Jang, Ganesh Sriram, Alisa Morss Clyne

**Affiliations:** 1 Fischell Department of Bioengineering, University of Maryland, College Park, MD 20742, USA; bmoiz@umd.edu (B.M.); asun42@umd.edu (A.S.); ali21029@umd.edu (A.L.); suryapad@umd.edu (S.P.); kalbus1@umd.edu (K.A.); 2 School of Bioengineering, Science, and Heath Systems, Drexel University, Philadelphia, PA 19104, USA; jonathangarcia.ua@gmail.com (J.G.); sarahbasehore92@gmail.com (S.B.); 3 Department of Biological Chemistry, Chao Family Comprehensive Cancer Center, University of California Irvine, Irvine, CA 92697, USA; choljang@uci.edu; 4 Department of Chemical and Biomolecular Engineering, University of Maryland, College Park, MD 20742, USA; gsriram@umd.edu

**Keywords:** metabolic flux analysis, fluxomics, endothelial metabolism, cardiovascular disease, polyol pathway, pentose phosphate pathway, hexosamine biosynthetic pathway, aldose reductase inhibitors

## Abstract

Disrupted endothelial metabolism is linked to endothelial dysfunction and cardiovascular disease. Targeted metabolic inhibitors are potential therapeutics; however, their systemic impact on endothelial metabolism remains unknown. In this study, we combined stable isotope labeling with ^13^C metabolic flux analysis (^13^C MFA) to determine how targeted inhibition of the polyol (fidarestat), pentose phosphate (DHEA), and hexosamine biosynthetic (azaserine) pathways alters endothelial metabolism. Glucose, glutamine, and a four-carbon input to the malate shuttle were important carbon sources in the baseline human umbilical vein endothelial cell (HUVEC) ^13^C MFA model. We observed two to three times higher glutamine uptake in fidarestat and azaserine-treated cells. Fidarestat and DHEA-treated HUVEC showed decreased ^13^C enrichment of glycolytic and TCA metabolites and amino acids. Azaserine-treated HUVEC primarily showed ^13^C enrichment differences in UDP-GlcNAc. ^13^C MFA estimated decreased pentose phosphate pathway flux and increased TCA activity with reversed malate shuttle direction in fidarestat and DHEA-treated HUVEC. In contrast, ^13^C MFA estimated increases in both pentose phosphate pathway and TCA activity in azaserine-treated cells. These data show the potential importance of endothelial malate shuttle activity and suggest that inhibiting glycolytic side branch pathways can change the metabolic network, highlighting the need to study systemic metabolic therapeutic effects.

## 1. Introduction

Endothelial cells play an important role in pathologies ranging from atherosclerosis to cancer to Alzheimer’s disease. Endothelial glucose metabolism is disrupted in many of these diseases. Recent efforts demonstrate that manipulating endothelial metabolism can alter cell signaling pathways and thereby cell function [1,2,3]. Many of these studies increased or decreased the activity of a single metabolic enzyme, often focused on glycolysis [4,5,6]. However, glucose metabolism involves a complex metabolic network, and disturbing one enzyme can potentially have effects on seemingly unrelated metabolic pathways. Glycolytic side branch pathways, including polyol, pentose phosphate, and hexosamine biosynthetic pathways, have been implicated in endothelial dysfunction in hyperglycemia; however, manipulating one of these side branch pathways could have unexpected effects on glycolysis, the tricarboxylic acid (TCA) cycle, or other glycolytic side branch pathways [7,8,9].

**Figure 1:**
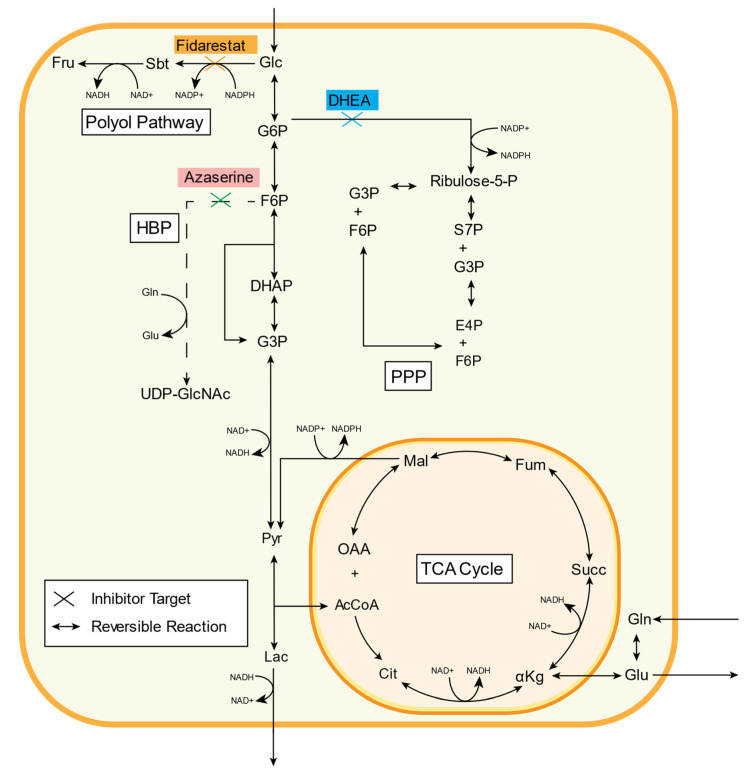
Three inhibitors (fidarestat, dehydroepiandrosterone (DHEA), and azaserine) were used to decrease glucose flux down glycolytic side branch pathways (polyol, PPP, and HBP, respectively). Fidarestat inhibits aldose reductase, an enzyme that catalyzes the first rate-limiting step of the polyol pathway and reduces glucose to sorbitol. DHEA inhibits glucose-6-phosphate dehydrogenase (G6PD), an enzyme involved in converting glucose-6P into 6P-glucolactone in the PPP. Azaserine inhibits the rate-limiting enzyme GFAT in the HBP, a pathway responsible for producing UDP-GlcNAc. See Table 1 for metabolite abbreviations.

Excess glucose enters the polyol pathway from glycolysis when hexokinase becomes saturated (Figure 1) [10]. Aldose reductase then catalyzes glucose reduction to sorbitol, oxidizing NADPH to NADP^+^ in the reaction. Sorbitol, a hydrophilic alcohol, cannot readily diffuse through the cell membrane, and its accumulation inside the cell potentially causes osmotic stress [11]. Sorbitol can also be oxidized to fructose, reducing NAD^+^ to NADH in this reaction. Fructose can then be converted to 3-deoxyglucosone (3-DG), which promotes formation of damaging advanced glycation end products (AGE) [12]. Increased polyol pathway activity can thereby alter the redox balance inside a cell by both decreasing the NAD^+^/NADH ratio and increasing the NADP^+^/NADPH ratio [13]. The polyol pathway has been implicated in diabetic retinopathy and neuropathy, endothelial cell dysfunction, and myocardial ischemia-reperfusion injury among others through activating proliferative signaling pathways and enhancing oxidative stress [7,14,15]. However, clinical trials of aldose reductase inhibitors showed modest effects on diabetic retinopathy and neuropathy [16,17].

Hyperglycemia can alter metabolic flux in the pentose phosphate pathway (PPP), which consists of oxidative and non-oxidative branches (Figure 1) [18]. The unidirectional oxidative branch consumes glucose 6-phosphate (G6P) following the first step of glycolysis. G6P is catalyzed to 6-phosphogluconolactone by G6P dehydrogenase (G6PD) and eventually to ribulose 5-phosphate, creating two NADPH from NADP^+^. Thus, the PPP oxidative branch produces NADPH, an essential reducing agent for fatty acid, sterol, nucleotide, and non-essential amino acid synthesis. The bidirectional non-oxidative branch converts ribulose 5-phosphate to ribose 5-phosphate, a nucleic acid building block, and xylulose 5-phosphate. These metabolites can then be converted into fructose 6-phosphate or glyceraldehyde 3-phosphate, re-entering glycolysis [19]. Oxidative PPP flux increases under oxidative stress, and overexpressing G6PD decreased reactive oxygen species and diabetic cardiomyopathy [20,21]. Non-oxidative PPP flux increases during cell proliferation. Both PPP branches are important in cancer and interact with oncogenic pathways (e.g., p53, HIF-1) [22].

Increased endothelial glucose flux also affects the hexosamine biosynthetic pathway (HBP), which is important in the post-translational protein modification O-GlcNAcylation (Figure 1) [23,24]. In the HBP, fructose-6-phosphate (F6P) is converted to glucosamine-6-phosphate (Glc6P) by the rate-limiting enzyme glutamine:fructose 6-phosphate amidotransferase (GFAT). Alternatively, exogenous glucosamine can enter the HBP at this step after conversion to Glc6P by hexokinase. Three subsequent reactions use acetyl-CoA and UTP to form UDP-N-acetylglucosamine (UDP-GlcNAc). UDP-GlcNAc then serves as a substrate for the O-linked β-N-acetylglucosamine (O-GlcNAc) modification of protein serine/threonine residues [25]. Protein O-GlcNAcylation is important in physiologic cell processes such as transcription, stress response, calcium cycling, and signaling, as well as in pathology [26,27]. Proteins are often O-GlcNAcylated at the same site where they are phosphorylated [28]. Thus, increased or decreased O-GlcNAcylation can impact intracellular signaling pathways.

The goal of this work was to study how inhibiting glycolytic side branch pathways using established chemical inhibitors would affect other parts of the metabolic network. Fidarestat, an aldose reductase inhibitor that has shown some promise in clinical trials of diabetic neuropathy, was used to inhibit the polyol pathway [29]. Dehydroepiandrosterone (DHEA), a sex hormone precursor that may also increase and activate endothelial nitric oxide synthase to regulate vascular function, was used to inhibit the PPP [30,31]. Azaserine, a structural glutamine analog that competitively inhibits reactions that involve glutamine, was used to inhibit the HBP (Figure 1) [32]. We analyzed the effects of these inhibitors on endothelial glucose metabolism via extracellular flux measurements and mass spectrometry. We then used metabolic flux analysis to estimate changes in metabolic fluxes with each inhibitor. We now show that these metabolic inhibitors can have systemic effects on endothelial cell metabolism, which we speculate may be due to either compensatory mechanisms or signaling pathway activation. This study thus suggests that the entire metabolic network should be analyzed when developing metabolic therapies.

## 2. Results

### 2.1. ^13^C Metabolic Flux Analysis of Baseline Endothelial Cell Metabolism

**Figure 2:**
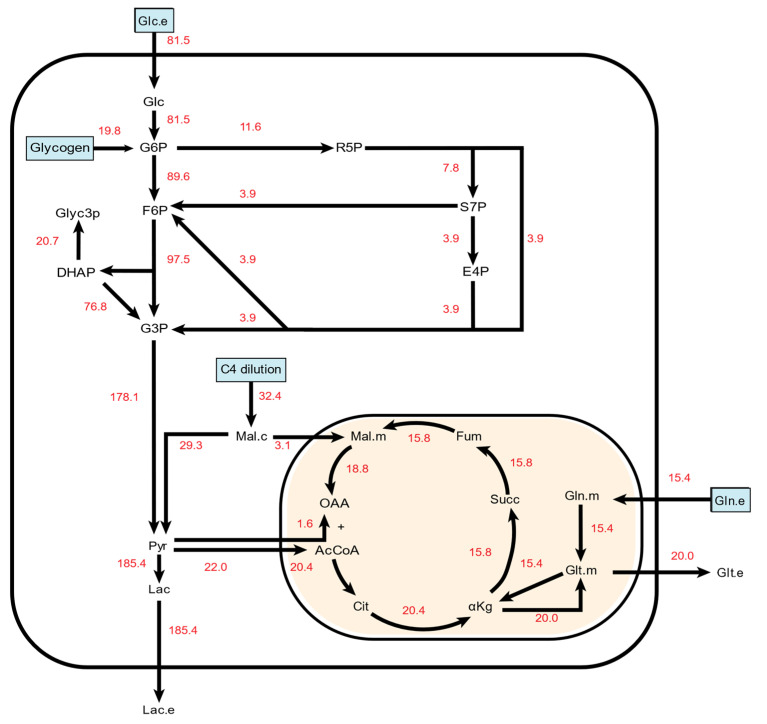
A simplified metabolic flux map depicting net metabolic fluxes in untreated HUVECs. All numbers are given in nmol/cells/hr. Carbon sources in the final model are indicated in light blue boxes. To simplify PPP pathway visualization, only the unique metabolites formed in each reaction are depicted along with the net PPP-based production of F6P and G3P. Full information on reaction schemes, atom transition specifications, and fluxes can be found in Supplemental File S1.

We started by creating a ^13^C metabolic flux analysis (^13^C MFA) model to enhance our analytic capabilities (Supplemental File S1). Through an iterative process, we modified model parameters until we achieved a satisfactory fit for the untreated endothelial cell mass spectrometry data. This allowed us to estimate metabolic flux values and gain unique insights into HUVEC metabolism that could not have been obtained from analyzing the input and output fluxes and metabolic isotopomer distributions alone. In the final baseline HUVEC ^13^C MFA model (Figure 2), TCA flux rates were estimated to be around 20% of the incoming glucose flux rate. The model also predicted influx of a four-carbon compound that fed into the malate shuttle and diluted cytosolic malate. The rate of this flux was approximately 40% of the glucose influx rate in the untreated cells. In comparison, the rate of glutamine intake was 19% of the glucose influx rate. Glutamate outflow from the TCA cycle was equivalent to pyruvate-derived acetyl-CoA influx into the mitochondria. The model also predicted net malate shuttle activity from the cytoplasm into the mitochondria. 86% of pyruvate was derived from G3P, which is sourced by glucose influx, glycogen breakdown, and the PPP. The remaining 14% was derived from malate. Approximately 11% of pyruvate entered the mitochondria, while the remainder was secreted as lactate. Roughly 11% of G6P was estimated to enter the PPP.

### 2.2. Inhibitor Effects on Endothelial Cell Glucose Metabolism

**Figure 3:**
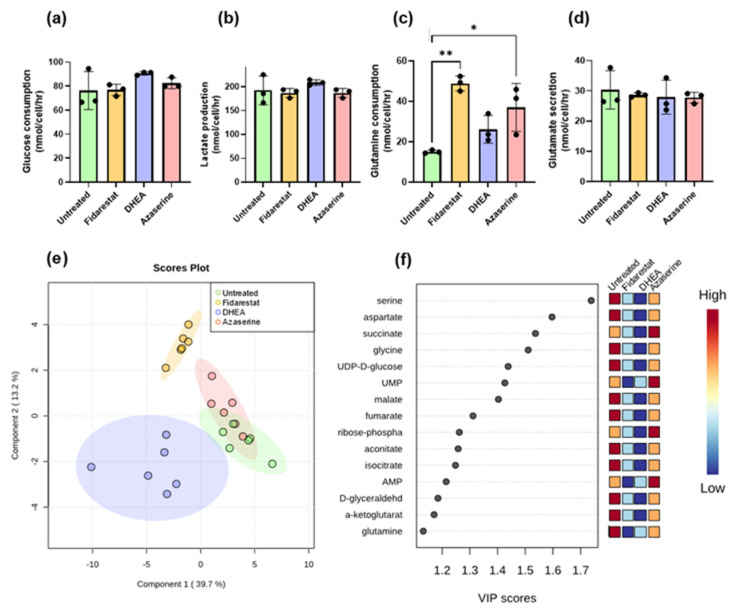
Fidarestat (aldose reductase inhibitor), DHEA (G6PD inhibitor), and azaserine (GFAT inhibitor) only changed external fluxes for glutamine consumption; however, fidarestat and DHEA-treated cells clustered separately from untreated cells by PLS-DA, with amino acids and TCA metabolites explaining most of the differences. (**a**) glucose consumption, (**b**) lactate secretion, (**c**) glutamine consumption, and (**d**) glutamate secretion all measured by YSI bioanalyzer after 24 h of inhibitor treatment. (**e**) PLS-DA clustering of total enrichment data. (**f**) VIP scores plot showing metabolites that significantly contributed to PLS-DA class discrimination. Results in (**a**–**d**) are mean +/− s.d. with n = 3 biological replicates. Results in e and f are n = 6 biological replicates. Statistical significance was determined with one-way ANOVA followed by Dunnett’s multiple comparison test. * *p* < 0.05; ** *p* < 0.01

We then examined the metabolite inputs (glucose, glutamine) and outputs (lactate, glutamine) for endothelial cells that were either untreated (vehicle control) or treated with each glycolytic side branch pathway inhibitor (Figure 3a–d, Supplemental File S2). Glucose consumption and lactate production both increased by 18% in endothelial cells treated with DHEA, and while this change was consistent across repeated experiments, high variability in untreated endothelial cell measurements prevented these differences from being statistically significant. The lactate/glucose ratio also decreased by 6–11% with all of the inhibitors, which again was consistent but not statistically significant (Supplemental Figure S2). Glutamine uptake more than tripled in endothelial cells treated with fidarestat and more than doubled in endothelial cells treated with azaserine. Glutamate secretion did not change, which meant that the glutamate/glutamine ratio decreased while the glutamine/glucose ratio increased (Supplemental Figure S2) in endothelial cells treated with azaserine and fidarestat.

We next used partial least squares discriminant analysis (PLS-DA) to reduce the dimensionality of the mass spectrometry dataset (Supplemental File S2) and select for metabolites and isotopomers that maximized differences among the treatment groups. Fidarestat- and DHEA-treated endothelial cells formed distinct clusters from untreated cells, whereas azaserine-treated endothelial cells overlapped with untreated cells (Figure 3e). The VIP scores showed that non-essential amino acids (serine, aspartate, glycine, glutamine) and TCA metabolites (succinate, malate, fumarate, aconitate, isocitrate, α-ketoglutarate) explained most of the variation among the samples (Figure 3f). PLS-DA analysis of individual isotopomers and subsequent VIP scores highlighted differential labeling among serine, glycine, succinate, and aspartate isotopomers (Supplemental Figure S3). UDP/UMP were also frequently represented among the top 50 scoring isotopomers (Supplemental File S3). On examining the total pools, the sizes of the HBP and PPP metabolite total pools were consistently lower in all treated samples, whereas TCA metabolite total pools were largely similar among untreated and treated samples (Supplemental Figure S8).

### 2.3. Fidarestat: Polyol Pathway Inhibition

**Figure 4:**
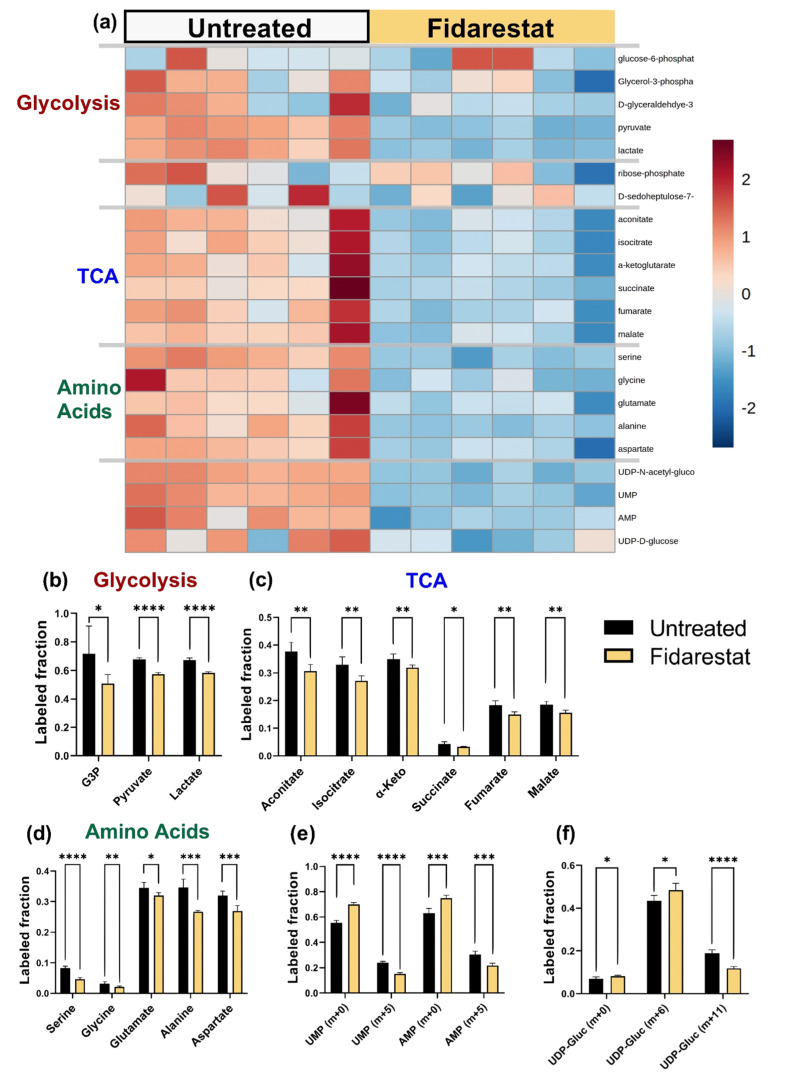
Fidarestat (aldose reductase inhibitor) decreased the ^13^C enrichments of HUVEC glycolytic and TCA metabolites, as well as amino acids, nucleotide precursors, and UDP-glucose. (**a**) Heatmap comparing ^13^C metabolite enrichment of untreated HUVECs and fidarestat-treated HUVECs. ^13^C enrichment of (**b**) glycolytic metabolites, (**c**) TCA metabolites, (**d**) amino acids, (**e**) AMP and UMP, and (**f**) UDP-glucose. Statistical significance for (**b**–**f**) determined through Welch’s unequal variances *t*-test; * *p* < 0.05; ** *p* < 0.01; *** *p* < 0.001; **** *p* < 0.0001. n = 6 biological replicates.

We then analyzed each inhibitor individually by examining labeled metabolite distributions followed by ^13^C MFA. While fidarestat has been widely shown to inhibit the polyol pathway, we could not detect polyol pathway inhibition directly since our mass spectrometry data did not include sorbitol and we were unable to detect sorbitol using a colorimetric assay. Fidarestat treatment decreased the total ^13^C enrichment of glycolytic and TCA metabolites, the amino acids serine, glycine, aspartate, and alanine, as well as UDP-D-glucose and UDP-GlcNAc (Figure 4a; full heatmap in Supplemental Figure S4a). Upon closer examination of glycolytic metabolites, we observed that ^13^C enrichment of pyruvate decreased by about 10% with fidarestat treatment (*p* < 0.001) while ^13^C enrichment of lactate decreased by about 8% (*p* < 0.001; Figure 4b). The ^13^C enrichment of all measured TCA metabolites decreased by 3–7% (*p* < 0.01 for all but succinate for which *p* < 0.05) with fidarestat treatment. The ^13^C enrichment of fumarate and malate was around half that of the other TCA metabolites (Figure 4c), and succinate was more than 90% unlabeled. While amino acid ^13^C enrichment was low, the ^13^C enrichment of serine decreased by 43% (*p* < 0.001) and glycine by 34% (*p* < 0.01). Glutamate enrichment decreased by 7%, alanine by 22%, and aspartate by 16% (Figure 4d).

Overall ^13^C enrichment of nucleotides UMP and AMP decreased in fidarestat-treated cells (Figure 4e), with most of this accounted for by a 9% reduction in UMP [m + 5] and AMP [m + 5]. Fidarestat further altered the isotopomer distribution of UDP-glucose, a nucleotide sugar that is a precursor to glycogen, sucrose, lipopolysaccharides, and glycosphingolipids. The overall ^13^C enrichment of UDP-glucose did not change significantly; however, UDP-glucose [m + 6] increased while UDP-glucose [m + 11] decreased with fidarestat treatment (Figure 4f).

**Figure 5:**
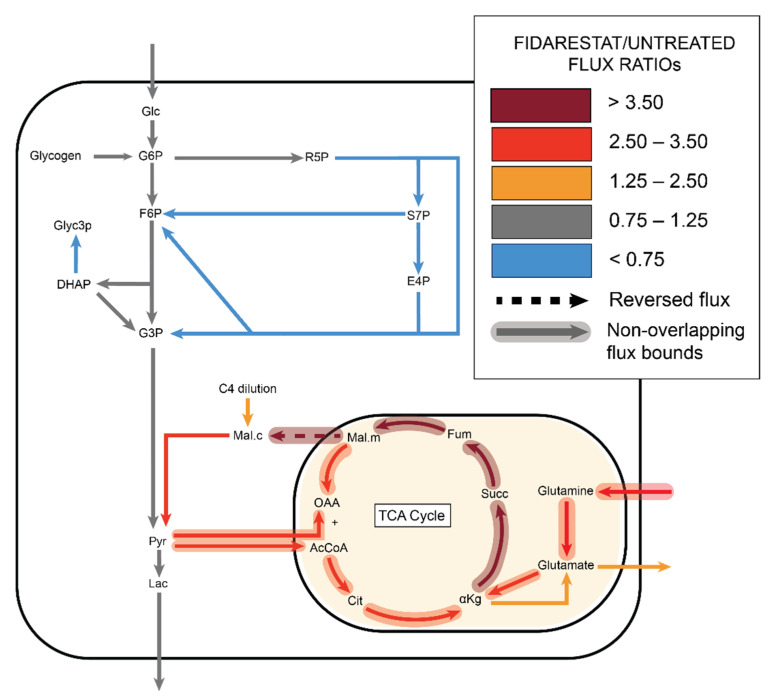
^13^C MFA estimated decreased non-oxidative PPP flux with increased TCA flux in fidarestat (aldose reductase inhibitor) treated cells, with the malate shuttle exporting malate from mitochondria to cytoplasm.

^13^C MFA of the fidarestat-treated endothelial cells predicted several changes in metabolic fluxes (Figure 5, Supplemental File S4). While glycolytic fluxes were estimated to be similar to the untreated group, the model predicted a decrease in non-oxidative PPP fluxes in the fidarestat treated group, although the 95% confidence intervals of these fluxes overlapped with the untreated group (Supplemental File S5). Fidarestat-treated cells were predicted to take in 31% more carbon than untreated cells, with 21% of the total carbon intake from glutamine and another 25% from the malate dilution flux. All TCA fluxes were predicted to increase significantly in fidarestat-treated cells, with no overlap in the 95% confidence intervals for the forward TCA fluxes between the treated and untreated groups. High glutamine intake in fidarestat-treated cells led to nearly four-fold increases in alphaketoglutarate dehydrogenase (AKG ➔ succinate), succinate dehydrogenase (succinate ➔ fumarate), and fumarase (fumarate ➔ malate) fluxes. Notably, the malate shuttle direction reversed from mitochondrial import to export with fidarestat treatment.

### 2.4. DHEA: Pentose Phosphate Pathway Inhibition

**Figure 6:**
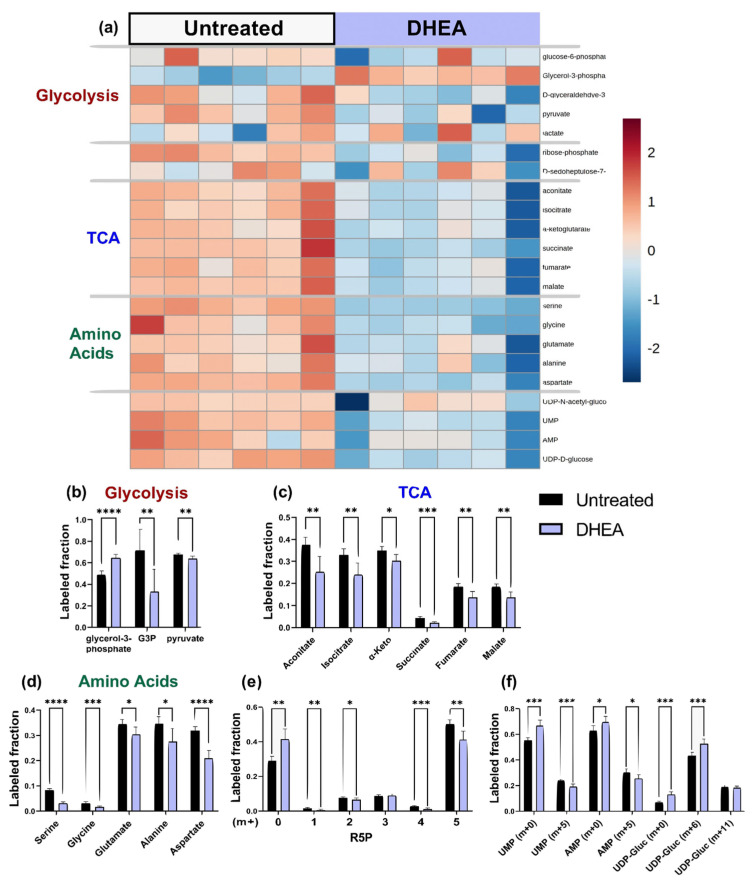
DHEA (G6PD inhibitor) decreased the ^13^C enrichments of HUVEC glycolytic and TCA metabolites, as well as amino acids, R5P, and nucleotide precursors UMP and AMP. (**a**) Heatmap comparing ^13^C metabolite enrichment of untreated HUVECs and DHEA-treated HUVECs. ^13^C enrichment of (**b**) glycolytic metabolites, (**c**) TCA metabolites, (**d**) amino acids, (**e**) R5P, and (**f**) UMP, AMP, and UDP-glucose. Statistical significance for (**b**–**f**) determined through Welch’s unequal variances *t*-test; * *p* < 0.05; ** *p* < 0.01; *** *p* < 0.001, **** *p* < 0.0001. n = 6 biological replicates.

We first confirmed that DHEA treatment of endothelial cells successfully reduced the activity of G6PD, the first and rate-limiting enzyme of the PPP, by 40% (Supplemental Figure S5a). DHEA had mixed effects on ^13^C enrichment of glycolytic metabolites; however, DHEA treatment consistently decreased ^13^C enrichment of TCA metabolites, amino acids, UDP-D-glucose, NAD, UMP, AMP, and the PPP metabolite ribose-5-phosphate (Figure 6a, full heatmap in Supplemental Figure S4b). When the ^13^C enrichment of glycolytic metabolites from DHEA-treated endothelial cells was examined in more detail, there was a 53% reduction in ^13^C enrichment of G3P (*p* < 0.001), as well as a slight decrease in pyruvate (Figure 6b). DHEA-treated cells showed 32% increased labeling of glycerol-3-phosphate, a dihydroxyacetone phosphate (DHAP) derivative. ^13^C enrichment of all measured TCA metabolites decreased by 10–32% with DHEA treatment (Figure 6c). ^13^C enrichment of serine decreased by more than 50% (*p* < 0.001) and ^13^C enrichment of glycine, glutamate, alanine, and aspartate also decreased in DHEA-treated cells (Figure 6d). In the PPP, the R5P isotopomer distribution changed in endothelial cells treated with DHEA. R5P [m + 0] increased by roughly 10%, while R5P [m + 5] decreased by nearly the same amount (Figure 6e). Finally, the unlabeled fractions of nucleotides UMP and AMP increased significantly, while the [m + 5] isotopes of these compounds decreased. UDP-glucose [m + 6] increased with DHEA treatment (Figure 6f).

**Figure 7:**
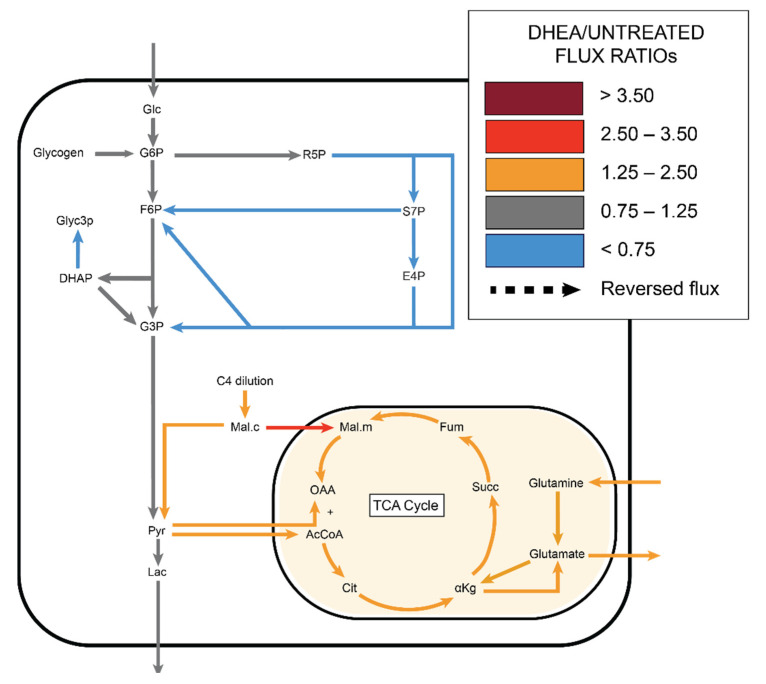
^13^C MFA estimated decreased non-oxidative PPP flux with increased TCA flux in DHEA (G6PD inhibitor) treated cells, with the malate shuttle importing malate from cytoplasm to mitochondria.

^13^C MFA predicted no change in glycolytic activity with DHEA. In the PPP, although the G6PDH reaction flux (G6P ➔ R5P) did not change, other PPP fluxes were estimated to decrease by 32%; however, they remained within the confidence interval of the untreated group. TCA cycle activity increased in endothelial cells treated with DHEA, although the lower bound of 95% confidence intervals for many of these fluxes slightly overlapped with the untreated group (Figure 7). Malate transport into the mitochondria increased by a factor of 3 (Supplemental File S4, Supplemental File S5).

### 2.5. Azaserine: Hexosamine Biosynthetic Pathway Inhibition

**Figure 8:**
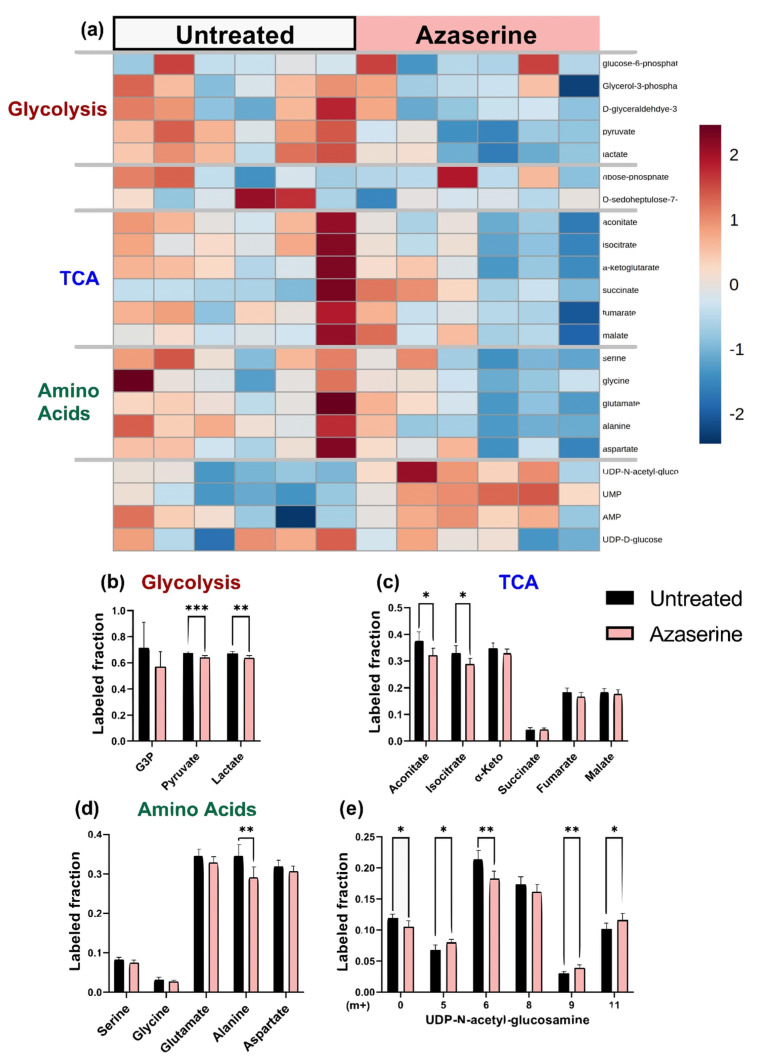
Azaserine (GFAT inhibitor) decreased the ^13^C enrichments of HUVEC glycolytic metabolites and had varied effects on TCA metabolites, amino acids, and UDP-GlcNAc. (**a**) Heatmap comparing ^13^C metabolite enrichment of untreated HUVECs and azaserine-treated HUVECs. ^13^C enrichment of (**b**) glycolytic metabolites, (**c**) TCA metabolites, (**d**) amino acids, and (**e**) UDP-GlcNAc. Statistical significance for (**b**–**f**) determined through Welch’s unequal variances *t*-test; * *p* < 0.05; ** *p* < 0.01; *** *p* < 0.001. n = 6 biological replicates.

Azaserine-treated endothelial cells, which should have decreased HBP flux, showed decreased UDP-GlcNAc in the total pool data (Supplemental Figure S8) and 30% lower protein O-GlcNAcylation by Western blot (Supplemental Figure S5b). Azaserine-treated cells appeared similar to untreated cells on the ^13^C enrichment heat map. Total glycolytic and TCA intermediate metabolites decreased slightly with azaserine treatment, while amino acids and nucleotides did not change consistently (Figure 8a, full heatmap in Supplemental Figure S4c). When ^13^C enrichments were analyzed in additional detail, ^13^C enrichment of glycolytic metabolites pyruvate and lactate decreased by 5% with azaserine treatment (*p* < 0.001 and *p* < 0.01, respectively; Figure 8b). Similarly, TCA metabolite ^13^C enrichment decreased by 4–15% with azaserine treatment, with only acotinate and isocitrate statistically significant (*p* < 0.05; Figure 8c). ^13^C enrichment of amino acids was similar between azaserine treated and untreated cells, except for alanine, which showed a 15% lower ^13^C enrichment in azaserine treated cells (Figure 8d). The isotopomer distribution of UDP-GlcNAc showed a different pattern with azaserine treatment, with UDP-GlcNAc [m + 0], [m + 6], and [m + 8] decreasing and UDP-GlcNAc [m + 5], [m + 9], and [m + 11] increasing for azaserine treated cells (Figure 8e). The largest change was in UDP-GlcNAc [m + 6], which decreased by 15% (*p* < 0.01).

**Figure 9:**
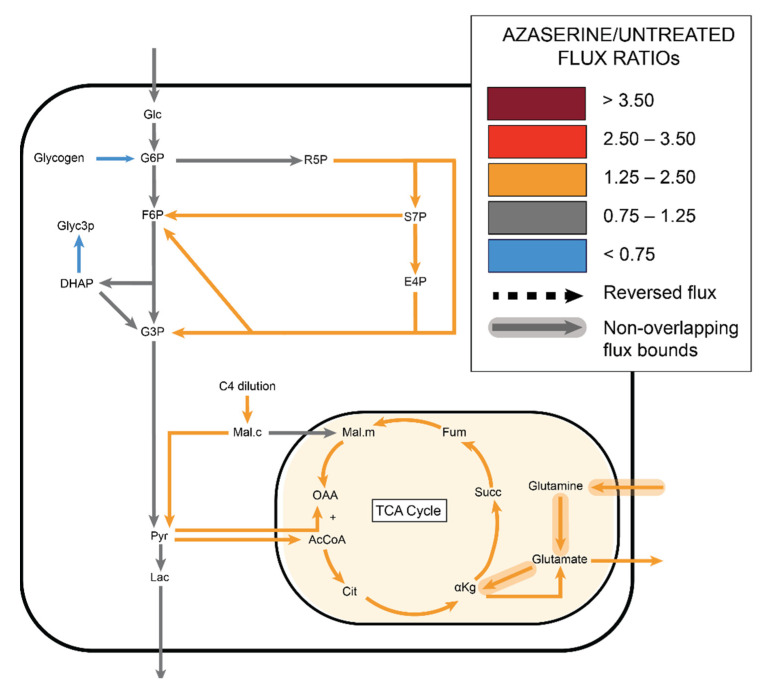
^13^C MFA estimated increased non-oxidative PPP flux with increased TCA flux in azaserine (GFAT inhibitor) treated cells.

Similar to the other groups, ^13^C MFA predicted no change in glycolytic flux with azaserine. However, in contrast to the other groups, MFA predicted that PPP flux would increase by 1.4–1.7 over fluxes in untreated cells, although the confidence intervals for these fluxes overlapped. ^13^C MFA further predicted increased flux throughout the TCA cycle, driven by a combination of increased pyruvate shuttling into the mitochondria, glutamine influx, and malate influx (Figure 9, Supplemental File S4, Supplemental File S5).

## 3. Discussion

Endothelial metabolic perturbations are implicated in vascular dysfunction and disease [2]. Understanding the systemic impact of metabolic inhibitors is crucial to characterizing their therapeutic efficacy and safety. In this study, we analyzed the effects of fidarestat (polyol pathway), DHEA (PPP), and azaserine (HBP) on the endothelial metabolic network. We now demonstrate that the impact of these metabolic inhibitors extends beyond their intended targets and may significantly perturb other metabolic pathways, particularly the TCA cycle. These analyses highlight the importance of integrated experimental and computational studies of the effects of pathway-specific inhibitors on the entire metabolic network.

Several unique metabolic characteristics of endothelial cells became evident in the baseline ^13^C MFA model [2]. An unlabeled four-carbon (C_4_) source flux was required to fit the ^13^C MFA model for all four treatment groups. We propose several possible explanations for the unlabeled C_4_ source (Supplemental Figure S6). First, the C_4_ compound could come from media proteins or amino acids. Glutamine (2 mmol/L) is the most concentrated media amino acid. When we simulated glutamine entry and cytosolic conversion to oxaloacetate in our model, we obtained a satisfactory fit (data not shown). However, these reactions typically occur in mitochondria and to our knowledge, there is limited evidence of that they occur in cytosol. Furthermore, this model predicted increased PPP flux in fidarestat-treated cells, which contradicts isotope labeling data.

An alternative unlabeled C_4_ source may be malate drawn from preexisting intracellular carbon pools such as cytoplasmic succinate or succinyl-CoA. Succinate accumulates in mitochondria when succinate dehydrogenase is inhibited [33]. Excess mitochondrial succinate is then transported into the cytosol by the dicarboxylate transporter SLC25A10 [34]. Succinyl-CoA could also be produced outside mitochondria [35]. In support of this hypothesis, succinate was 95–97% unlabeled in all samples whereas other TCA metabolites were 60–80% unlabeled. Our ^13^C MFA metabolic model predicted that 85–91% of succinate is in the cytoplasm. Cytosolic succinate is known to affect cell signaling by promoting protein succinylation and impact the epigenetic landscape by inhibiting histone and DNA methylases [36,37]. Thus, high cytosolic succinate may identify an important connection between endothelial cell metabolism and signaling pathways. However, the amount of preexisting unlabeled succinate may be too small to account for the substantive C_4_ influx predicted by ^13^C MFA.

Finally, the arginine/citrulline cycle may also be an unlabeled C_4_ source. Arginine, which is present in the medium, is converted to citrulline by endothelial nitric oxide synthase (eNOS) to generate nitric oxide (NO). When citrulline and aspartate react to form fumarate and recycled arginine, the cytosolic fumarate can then be converted into malate [38]. The reaction requires aspartate import or synthesis, which may come from degradation of media threonine or other aspartate-family amino acids. This hypothesis is supported by the increased dilution of fumarate and malate as compared to other TCA metabolites in the isotopomer data. In addition, endothelial cells have several arginine transporters and retain high cytoplasmic arginine for continuous NO production [39]. Glutamine, succinate, and arginine are likely not mutually exclusive but rather their combination provides the additional C_4_ sources.

Fidarestat significantly impacted core metabolic pathways, even though the polyol pathway is considered primarily active in hyperglycemia [7]. Our ^13^C MFA model suggests that increased glutamine rather than glucose uptake provides carbons for the higher TCA activity, leading to TCA metabolite dilution [40]. This prediction is supported by our observation that mitochondrial potential increases in fidarestat-treated HUVEC (Supplemental Figure S10). Fidarestat may affect glutamine uptake and TCA activity via sirtuin 1 (SIRT1), an NAD^+^-dependent deacetylase that regulates metabolic gene expression and may be protective in vascular tissue [41,42]. SIRT1 promotes glutamine uptake by enhancing reductive carboxylation through increased glutamine transporter SLC1A5 and mitochondrial glutaminase isoform 1 (GLS1) expression [43]. Fidarestat increased SIRT1 expression in both HUVEC in vitro and diabetic mice in vivo [44]. Since SIRT1 activity also depends on the NAD^+^/NADH ratio, fidarestat may also increase SIRT1 activity since less NAD^+^ is converted to NADH when the polyol pathway is inhibited [45,46]. Alternatively, fidarestat may regulate glutamine uptake and TCA activity via nuclear factor erythroid 2-related factor 2 (Nrf-2), a transcription factor important in oxidative stress response. An aldose reductase inhibitor increased Nrf-2 activity in colon cancer cells [47], and Nrf-2 increased glutamine uptake and metabolism in murine cells [48,49].

Fidarestat may decrease PPP flux through the intracellular NADP^+^/NADPH balance. We measured a lower NADP^+^/NADPH ratio in fidarestat-treated as compared to untreated cells (Supplemental Figure S9), likely since aldose reductase inhibition reduced NADPH oxidation to NADP^+^. Increased cytosolic malate in fidarestat-treated endothelial cells can be converted into pyruvate and NADPH via malic enzyme. We hypothesize that increased TCA activity with fidarestat also increased malic enzyme activity, resulting in increased cytosolic NADPH production [22,50]. The malic enzyme-produced NADPH, along with decreased NAPDH consumption in the polyol pathway, may then increase NADPH to allosterically inhibit PPP flux [51]. An effect of decreased non-oxidative PPP activity in fidarestat-treated endothelial cells is reduced formation of the five-carbon ribose-5-phosphate (R5P), which is observed in decreased ^13^C enrichment of UMP [m + 5], AMP [m + 5], and amino acids. Endothelial cells synthesize or take up serine for nucleotide synthesis; thus, decreased nucleotide synthesis via the PPP may require increased serine import from the media [52,53]. Increased serine influx would decrease ^13^C enrichment of serine and its derivative, glycine, as well as G3P and pyruvate. In addition, decreased PPP flux may increase G6P [m + 6] in the cytosol and thus route G6P down alternate pathways such as UDP-glucose synthesis from G6P-derived G1P and UMP. Increased G6P [m + 6] combined with decreased UMP [m + 5] could lead to the observed increased UDP-glucose [m + 6] and decreased UDP-glucose [m + 11] (Supplemental Figure S7a).

Although endothelial cells treated with the PPP inhibitor DHEA were similar to fidarestat-treated cells, these changes likely occurred through a reversal of the same mechanism. We measured an increased NADP^+^/NADPH ratio in DHEA-treated as compared to untreated cells (Supplemental Figure S9), which fits with decreased NADPH reduction to NADP^+^ when G6PD is inhibited. To compensate for decreased PPP activity and therefore reduced NADPH production, DHEA-treated endothelial cells may have increased glutamine intake and malic enzyme activity to restore NADPH. These cells may have also increased serine consumption to drive nucleotide synthesis. Studies in mouse oocytes suggest that DHEA inhibition of PPP may motivate citrate conversion to malate to produce NADPH and thereby restore reduction as well as fatty acid, cholesterol, and steroid hormone production [54]. DHEA treatment in primary corneal fibroblasts increased isocitrate, fumarate, and malate [55]. Endothelial cells have also been shown to sustain TCA activity for nucleotide synthesis [2]. TMRM staining demonstrated increased mitochondrial potential in DHEA-treated cells (Supplemental Figure S10). Increase TCA cycle activity in our study may indicate a compensatory mechanism in DHEA-treated endothelial cells.

Azaserine inhibition of the HBP slightly increased PPP activity, which could be due to cytosolic F6P and hence G6P accumulation. Another possibility is that azaserine decreases PPP inhibition [56]. Glucosamine-6-phosphate (GlcN6P), which is produced in the first HBP reaction, inhibited the PPP via G6PD inhibition in bovine venular endothelial cells and human embryonic stem cells [57,58]. Thus, reduced GlcN6P in azaserine-treated cells may in turn reduce G6PD inhibition and increase PPP flux. However, we measured an increased NADP^+^/NADPH ratio in azaserine-treated as compared to untreated cells (Supplemental Figure S9). Since azaserine inhibits reactions that involve glutamine, it is possible that there is another inhibited reaction that is causing this effect. We also observed increased mitochondrial potential in azaserine-treated cells (Supplemental Figure S10). Increased TCA activity with decreased TCA metabolite ^13^C enrichment with azaserine treatment could relate to increased glutamine uptake as the endothelial cells tried to overcome azaserine competitive inhibition of glutamine reactions. Interestingly, azaserine treatment did not change the overall ^13^C enrichment of UDP-GlcNAc, the primary HBP endpoint, but instead shifted isotopomer labeling. Azaserine reduced UDP-GlcNAc [m + 6] and [m + 8], which represent decreased labeled F6P incorporation into UDP-GlcNAc through the HBP. UDP-GlcNAc [m + 5] and UDP-GlcNAc [m + 11] increased, likely due to increased UDP [m + 5] (Supplemental Figure S7b).

While to the best of our knowledge this is the first application of ^13^C MFA to study HUVEC metabolism, our study is not without limitations. We present hypotheses supported by the model, along with some validation studies; however, further in vitro and in vivo studies are needed to validate these hypotheses. We did not consider the impact of insulin, which may have been present in our culture medium since endothelial cells primarily use the insulin-independent glucose transporter GLUT1. All of the inhibitors in our studies have off-target effects which may or may not relate to metabolism [59]. ^13^C MFA quantified fluxes in core metabolic pathways; however, the model could not resolve fluxes in peripheral pathways such as HBP and UDP-glucose since these pathways terminated in sink nodes that did not tie back to core metabolic pathways.

Together, our combined experimental and computational approach highlights potential systemic effects of metabolic inhibitors on endothelial metabolism. We show that all three inhibitors increased TCA activity, suggesting that the TCA cycle attenuates perturbed endothelial metabolism. In addition, carbon influx prior to malate or oxaloacetate appears to be a crucial aspect of HUVEC metabolism. Metabolic therapeutics are increasingly explored as treatments for cancer [60] and cardiovascular disease [61] among others. However, the interconnected nature of metabolism means that these therapeutics need to be closely examined to reduce off-target effects prior to clinical implementation.

## 4. Materials and Methods

### 4.1. Cell Culture and Pathway Inhibition

Human umbilical vein endothelial cells (HUVEC, Cell Applications) were cultured in a 37 °C, 5% CO_2_ incubator in Endothelial Growth Medium-2 (EGM-2; Lonza) supplemented with 10% fetal bovine serum (FBS; HyClone), 1% penicillin-streptomycin, and 1% L-glutamine (Gibco). Media was changed every two days, and HUVEC were used between passages 4 and 8.

The polyol pathway, PPP, and HBP were inhibited using fidarestat, DHEA, and azaserine respectively. For inhibitor studies, HUVEC were seeded near confluence and incubated for 48 h until cells formed a confluent monolayer. Media was then changed to Endothelial Basal Medium-2 (EBM-2; Lonza) supplemented with 10% FBS, 1% penicillin-streptomycin, and 1% L-glutamine (Gibco) and either 18 µM fidarestat; 5 µM DHEA; or 25 µM azaserine (Cayman Chemical) was added for 24 h. Untreated cells were cultured in 0.1% dimethylsulfoxide (DMSO) as a vehicle control. Dose-response studies confirmed that 50 µM DHEA inhibited G6PDH activity (Abcam) in HUVECs by about 40% (Supplemental Figure S5a), and 25 µM azaserine significantly reduced protein O-GlcNAcylation by Western blot (Supplemental Figure S5b). No significant cell death was observed at these inhibitor concentrations.

### 4.2. Isotope Labeling and Mass Spectrometry

Endothelial glucose metabolism was measured via YSI bioanalysis and liquid chromatography mass spectrometry (LC-MS). YSI was used to measure glucose uptake and lactate output, as well as glutamine uptake and glutamate output. For YSI, 200 μL conditioned media was collected from each replicate and pipetted into a 96-well plate for analysis by a YSI Bioanalyzer 2950.

For mass spectrometry, HUVEC were cultured with 5 mM U-^13^C_6_-glucose (Cambridge Isotope Laboratories) in DMEM without glucose, glutamine, and pyruvate (ThermoFisher Scientific) supplemented with 10% dialyzed FBS, 1% penicillin-streptomycin, and 1% L-glutamine for 24 h, as prior studies indicated that cells reached isotopic steady-state by this time. The medium was then removed and 80:20 methanol:water (−80 °C, extraction solvent) was added to cells for 15 min at −80 °C. Cells were scraped in the extraction solvent and cell lysates pipetted into Eppendorf tubes. Samples were centrifuged at 16,000 g for 10 min at 4 °C to pellet debris. The supernatant was transferred to a new tube, desiccated under nitrogen gas flow, and re-dissolved in LC-MS grade water. Metabolites were analyzed via reverse-phase ion-pairing chromatography coupled to an Exactive Orbitrap mass spectrometer (ThermoFisher, San Jose, CA, USA) following an established protocol [62]. Correction for natural abundance was performed using previously described protocols [63].

### 4.3. Metabolic Flux Analysis (MFA)

Metabolic flux analysis (MFA) combines experimental measurements and mathematical modeling to predict intracellular metabolic fluxes [64]. MFA requires a metabolic map that includes all reactions of interest, as well as experimental external metabolite rates and isotope-labeled metabolite distributions [64,65]. MFA then integrates these inputs to infer metabolic fluxes.

First, a metabolic network map was manually constructed to include relevant metabolic reactions, including glycolysis, the TCA cycle, and glycolytic side branches of interest (polyol pathway, PPP, HBP; Supplemental File S1) [66,67]. The metabolic network included dilution and pseudo reactions to account for unlabeled carbon sources and compartmentalization, respectively. Next, external metabolite rates (r_i_; nmol/10^6^ cells/h) for each metabolite (glucose, glutamine, lactate, glutamate) were calculated for non-proliferating cells from the YSI data as [64]:

$$r_{i}=1000*\;\frac{∆C_{i}*V}{∆t*N_{x}}$$

where *V* is volume (mL); ∆*C_i_* is metabolite concentration change between the two measurement times; ∆*t* is time (hours); and *N_x_* is cell number (2.2 million cells for a confluent 6-well plate). Finally, the isotope-labeled metabolite distributions from the mass spectrometry data were incorporated into the mass distribution vector (MDV), which describes the fractional abundance of each potential labeled isotope (from m + 0 with no labeled carbons to m + n with fully labeled carbons) [65,68]. The experimental standard deviation was selected as the maximum value between the actual experimental error or 1%, the standard error rate for LC-MS experiments [64].

Steady-state metabolic fluxes were then predicted using the MATLAB-based software package INCA [69], which uses optimization to identify a flux distribution that minimizes the sum of squared residuals (SSR) between simulated and experimentally derived MDVs. Flux values were determined using a Levenberg–Marquardt gradient descent algorithm. To improve the odds of finding the global optimum, simulations were repeated 100 times, each time beginning from a random initial point. The goodness of fit is based on the assumption that the minimized SSR follows a chi-square distribution. An acceptable fit is based on the n–p degrees of freedom, where n is the number of independent measurements and p is the number of fitted parameters. The formula for the expected SSR range is $\chi_{\alpha /2\;}^{2}$(n − p), $\chi_{1-\alpha /2\;}^{2}$(n − p)). The metabolic network map was iteratively modified, and the map and flux values that resulted in the minimum SSR were retained as the most likely flux distribution.

Reactions from glycolysis, TCA cycle, PPP, and HBP were used to build the model. Metabolites with missing or poor-quality mass spectrometry data were either (i) excluded from the model or (ii) lumped with adjacent metabolites if there were no changes in carbon arrangement. Both glucose and glutamine were essential carbon inputs to the model. However, we were not able achieve a satisfactory fit with these carbon sources alone (SSR = 220). A G6P dilution term representing glycogen breakdown was added to the model to account for unlabeled G6P. The glycogen source reaction improved model fit, but the overall SSR of 178 did not satisfy the acceptable range of [27.6, 64.2]. However, additional unlabeled carbons were needed to fit m + 0 and m + 2 malate, alpha ketoglutarate, and glutamate as well as the absence of m + 1 and m + 2 pyruvate and lactate in the experimental data. We therefore added an unlabeled three-carbon source to dilute pyruvate and a four-carbon source to dilute malate. Incorporating the three-carbon dilution to pyruvate slightly decreased the SSR value to 166, while the four-carbon dilution to malate reduced the SSR to 42, which was considered an acceptable fit (Supplemental Figure S1).

The final ^13^C MFA HUVEC model consisted of 79 reactions, 105 fluxes, 39 metabolites, and 45 degrees of freedom (Supplemental Figure S1). Labeled glucose accounted for 60% of incoming carbon, while glutamine (9%), glycogen (15%), and C4 dilution (16%) accounted for the remaining unlabeled carbon. Several assumptions were made during model construction. Mitochondrial and cytoplasmic malate, succinate, alpha ketoglutarate, pyruvate, citrate, glutamate, and aspartate pools were assumed to mix during metabolite extraction. HBP reactions and data were successfully fitted in the final model. However, estimated values for HBP fluxes were close to zero (<10^−6^) in the untreated group and were associated with large uncertainty in all groups, likely due to the number of unbounded dilution reactions tied into this pathway. For this reason, HBP fluxes were excluded from our flux ratio analysis. The metabolic model, confidence intervals, and MFA results can be found in Supplemental File S1.

### 4.4. Statistical Analysis

Supervised partial least-square discriminant analysis, principal component analysis, and variable importance projection (VIP) scores were obtained using MetaboAnalyst [70]. Statistical analysis and visualization of external flux and isotope labeling experiments were performed with GraphPad Prism v9.0.0. Each treatment group in the mass spectrometry dataset consists of n = 6 biological replicates from one experiment. External flux measurements quantified by YSI were repeated 3 times and a representative dataset is shown. All error bars represent SD. Statistical tests for experimental data are specified under each figure. For ^13^C MFA, 95% confidence intervals were calculated using parameter continuation, which varies all parameters upward and downward one at a time until the chi-square distributions are reached.

## Figures and Tables

**Table 1:** Metabolite abbreviations.

Abbreviation	Full Name
Glc	Glucose
Sbt	Sorbitol
Fru	Fructose
G6P	Glucose-6-phosphate
R5P	Ribose 5-phosphate
S7P	Sedoheptulose
G3P	Glyceraldehyde
E4P	Erythrose 4-phosphate
F6P	Fructose 6-phosphate
Xu5P	Xylulose 5-phosphate
UDP-GlcNAc	Uridine diphosphate-N-acetylglucosamine
FDP	Fructose 1,6-bisphosphate
DHAP	Dihydroxyacetone phosphate
PEP	Phosphoenolpyruvate
Pyr	Pyruvate
Lac	Lactate
OAA	Oxaloacetate
AcCoA	Acetyl-CoA
Cit	Citrate
Cis	Cis-Aconitrate
Iso	Isocitrate
αKg	A-Ketoglutarate
Glu	Glutamate
Gln	Glutamine
Succ	Succinate
Fum	Fumarate
Mal	Malate

## Data Availability

The data presented in this study are available in Supplemental Files S1–S5.

## Supplementary Materials

The following are available online at https://www.mdpi.com/article/10.3390/metabo11040226/s1. Figure S1: A C4 dilution was needed to fit the model to the isotopomer data. Malate and α-ketoglutarate were major sources of error, particularly in the m + 0 and m + 2 isotopomers. After adding the C4 dilution, the model was able to reach a satisfactory fit. Figure S2: External flux rate ratios show changes in both glutamine/glucose and glutamate/glutamine ratios with inhibitors. (a) Lactate secretion/glucose consumption ratio, (b) glutamine/glucose ratio, and (c) glutamate/glutamine ratio. Mean +/− s.d. with n = 3 biological replicates. Statistical significance was determined with one-way ANOVA followed by Dunnett’s multiple comparison test; *, *p* < 0.05; **, *p* < 0.01; *** *p* < 0.001. Figure S3: PLS-DA of individual isotopomers confirmed that fidarestat and DHEA-treated cells clustered separately from untreated cells. (a) PLS-DA clustering of individual isotopomers and (b) VIP score plot showing top 25 isotopomers that significantly contribute to class discrimination. Figure S4: Heatmap comparing total enrichment of all metabolites in untreated HUVECs and HUVECs treated with (a) fidarestat (b) DHEA or (c) azaserine. Figure S5: Preliminary experiments demonstrated that the inhibitors affected the PPP and HBP. (a) G6PD activity in DHEA-treated HUVEC and (b) protein O-GlcNAcylation in azaserine-treated HUVEC. Figure S6: Potential pathways that explain the C_4_ influx predicted by the ^13^C MFA model. One possibility is that amino acids such as glutamine contribute carbons that directly form malate or indirectly form malate by first forming aspartate that is consumed in the arginine-citrulline-NO cycle. Another possibility is that the unlabeled succinate pool observed in our study may be metabolized to fumarate and malate. Figure S7: Changes in isotopomer distributions of (a) UDP-Glucose following NADPH allosteric inhibition of G6PD and (b) UDP-GlcNAc following azaserine inhibition. Isotopomer fractions are not independent. As labeled fractions increase, unlabeled fractions must decrease. Red arrows designated increased representation of isotopomer in mass distribution vector, while blue arrows designated decreased representation of isotopomer in mass distribution vector. Dotted lines represent isotopomers generated from unlabeled sources, such as glycogen-derived G1P or removed O-GlcNAc residues which can also act as a source. If flux towards a labeled isotopomer increases, the relation ratio of its unlabeled counterpart will decrease. Figure S8: Total pool data for (a) isocitrate, (b) α ketoglutarate, (c) succinate, (d) fumarate, (e) malate, (f) aspartate, (g) ribose-5-phosphate, (h) sedoheptulose-7-phosphate, (i) UDP-GlcNAc, (j) AMP, and (k) UMP in HUVEC treated with fidarestat, DHEA, or azaserine. Statistical significance was determined using ANOVA and Dunnett’s multiple comparison test to compare treated groups to control with significance shown as *** *p* < 0.001; ** *p* < 0.01; * *p* < 0.05. n = 6 biological replicates. Figure S9: Comparison of NADP^+^/NADPH ratio in HUVEC treated with fidarestat, DHEA, or azaserine. Statistical significance was determined using student’s *t*-test to compare treated groups to control with significance shown as *** *p* < 0.001; ** *p* < 0.01; * *p* < 0.05. n = 6 biological replicates. Figure S10: Comparison of TMRM/Mitotracker colocalization in HUVECs treated with fidarestat, DHEA, or azaserine. B) Colocalization was quantified with ImageJ and the JACoP software package. Statistical significance was determined by using student’s *t*-test to compare treated groups to control with significance shown as *** *p* < 0.001; ** *p* < 0.01; * *p* < 0.05. File S1: Baseline HUVEC ^13^C MFA model reaction schemes, atom transition specifications, and fluxes. File S2: External flux rates, raw and INCA formatted mass spectrometry data. File S3: Isotopomer scores from PLS-DA. File S4: ^13^C MFA flux values, ratios, and compartmentalization for untreated and DHEA-, fidarestat, and azaserine-treated cells. File S5: ^13^C MFA 95% confidence intervals for untreated and DHEA-, fidarestat, and azaserine-treated cells.
