# Supplementary material for: 13C Metabolic Flux Analysis Indicates Endothelial Cells Attenuate Metabolic Perturbations by Modulating TCA Activity
Source: Metabolites. 2021 Apr 7;11(4):226. doi: 10.3390/metabo11040226 (PMC8068087; doi:10.3390/metabo11040226)
Supplement: Supplementary file 1 [file metabolites-11-00226-s001.zip › HuVEC inhibitor Supplemental Methods.docx]

**Supplemental Methods**

*NADP+/NADPH ratio measurement*

HUVECs were seeded in a 96-well plate and allowed to grow until confluence. Upon reaching confluence, cells were treated with the relevant inhibitors for 24 hours. Following treatment, cells were lysed and incubated with reagents from an NADP+/NADPH kit (Promega), as per the kit protocols. Briefly, media was aspirated from each well and replaced with 50 uL PBS. Another 50 uL of pepared NADP+/NADPH reagent was added to each well. The plate was placed on a plate shaker for 1 minute to lyse cells then incubated at room temperature for 60 minutes. The reagent/cell lysis mixture was then transferred over to a white-walled 96-well plate. Relative luminescence levels were quantified with a Tecan Spark Multimode Microplate Reader.

*G6PD Assay*

Cells were seeded at 300,000 cells/ml in EGM and allowed to grow for 48 hours until cultures become confluent. Cell media was replenished with 3 mL EBM containing 10% FBs, 1%PSG  +/- 0, 5, 25, 50, 75, or 100 uM DHEA. Cells were incubated for 24 hours in this media. Following treatment, cells were washed with cold PBS. Following aspiration, another 100 uL of PBS was added to each sample. Cells were scraped into pre-chilled Eppendorf tubes and needle lysed using 25GA needles. The mixture was incubated at 4C for 10 minutes under rotation. Cells were then centrifuged at max speed (13,000 RPM) for 5 minutes at 4C. Supernatant was collected and 50 uL of supernatant was then added to a 96-well plate in duplicate. G6PD activity assay working buffer (Abcam) was then added to each well. Plates were allowed to incubate for 30 minutes. Fluorescent signal was quantified at 450 nm.

*TMRM Mitochondrial potential assay assay*

Cells were seeded at 100,000 cells/plate in glass-bottom dishes and allowed to grow for 48 hours. Following treatment, the media was replaced with EBM containing 1:1000 TMRM (ThermoFisher) and 1:5000 Green Mitotracker (Thermofisher) and incubated for 30 minutes at 37C. The media was replaced with fresh EBM-2 and cells were allowed to incubate for another hour. Cells were then imaged using a revolve microscope. Images were analyzed and quantified in ImageJ using the JaCoP package.(Bolte and Cordelières, 2006)

*Western blot*

Cells were treated with EBM containing 0, 5, or 25 uM Azaserine for 24 hours. Following treatment, cells were washed twice with cold PBS and lysed with a mixture of RIPA Buffer (Thermo Scientific Pierce), Halt Protease Inhibitor Cocktail (Thermo Scientific), and EDTA (Thermo Scientific). Cells were scraped to collect lysate and then incubated on a rotator for 10 minutes, vortexed, and centrifuged at 13,300g for 15 minutes at 4°C. Supernatant containing protein was collected and normalized by BCA Assay (Thermofisher) to ensure 3.5 ug of protein per lane. Samples were denatured by heating at 70°C, separated via SDS-PAGE on a 4-12% Bis-Tris gel (Thermofisher), and transferred to a PVDF membrane via the iBlot 2 Dry Blotting System (Thermofisher). Membranes were then blocked in 5% bovine serum albumin (BSA, Sigma-Aldrich), washed with PBS-T (Thermofisher), and incubated with primary antibodies for O-GlcNac, (1:1000, Cell signaling) B-actin (1:1000, Santa Cruz). Following primary staining, blots were washed with PBS-T, and incubated with corresponding secondary conjugated antibody (1:20000, Promega) for 2 hours at room temperature. Blots were washed with PBS-T again and then incubated with signal enhancer (Thermo Scientific Pierce) before being visualized with a Fluourchem digital imager (Alpha Innotech). Band intensity was quantified with AlphaView SA software
